# Social bonding: regulation by neuropeptides

**DOI:** 10.3389/fnins.2014.00171

**Published:** 2014-06-24

**Authors:** Claudia Lieberwirth, Zuoxin Wang

**Affiliations:** ^1^Department of Behavioral Science, Utah Valley UniversityOrem, UT, USA; ^2^Department of Psychology and Program in Neuroscience, Florida State UniversityTallahassee, FL, USA

**Keywords:** pair bond, affiliation, social recognition, oxytocin, vasopressin

## Abstract

Affiliative social relationships (e.g., among spouses, family members, and friends) play an essential role in human society. These relationships affect psychological, physiological, and behavioral functions. As positive and enduring bonds are critical for the overall well-being of humans, it is not surprising that considerable effort has been made to study the neurobiological mechanisms that underlie social bonding behaviors. The present review details the involvement of the nonapeptides, oxytocin (OT), and arginine vasopressin (AVP), in the regulation of social bonding in mammals including humans. In particular, we will discuss the role of OT and AVP in the formation of social bonds between partners of a mating pair as well as between parents and their offspring. Furthermore, the role of OT and AVP in the formation of interpersonal bonding involving trust is also discussed.

## Introduction

Lasting, positive, and affiliative social relationships (e.g., among spouses, family members, and friends) play an essential role in human society (for review see Baumeister and Leary, 1995). These relationships affect psychological, physiological, and behavioral functions (for review see Baumeister and Leary, 1995). In particular, the enduring attachments between socio-sexual partners (e.g., marital relationships) seem to have profound impacts on the cognitive, social, emotional, and physical well-being. For example, social bonds that provide a sense of social belonging (social connectedness) seem to protect against feelings of loneliness, depression, and even anxiety (Lee and Robbins, 1998, 2000; Williams and Galliher, 2006). Furthermore, stable and positive marital relationships have been implicated to increase the life expectancy compared to that of non-attached singles (House et al., 1988; Lillard and Waite, 1995; Drefahl, 2012). There are also strong positive associations between high levels of marital satisfaction and immune function as well as cardiovascular health (Kiecolt-Glaser and Newton, 2001; Kiecolt-Glaser et al., 2010). Close parent relationships positively impact the psychological well-being of both parents and their children (Silverstein and Bengtson, 1991; Graziano et al., 2009). The presence of positive, secure attachments between parents and children provide protection against depression and anxiety in the child as well as lead to higher levels of child academic success (Bögels and Phares, 2008; Sarkadi et al., 2008; Graziano et al., 2009).

On the contrary, the disruption of social bonds (e.g., caused by marital problems, confrontations, isolation, or neglect) can have a negative impact on mental and physical health (Steptoe, 1991; Curtis, 1995). For example, low levels of intimacy between partners as well as perceived loneliness have been associated with negative psychological states, such as depression and depressive symptoms (Kiecolt-Glaser and Newton, 2001; Alpass and Neville, 2003; Adams et al., 2004). In addition, relationship conflict and interpersonal stress are associated with alterations in immune function (Kiecolt-Glaser and Newton, 2001; Kiecolt-Glaser et al., 2010; Jaremka et al., 2013). Similarly, adverse childhood experiences (such as parental separation or divorce, abuse, or neglect) have detrimental effects on the child's cognitive, physical, social, and emotional well-being. Early adverse childhood experiences are associated with poor child health at an early age (e.g., increased risk for asthma as well as cardio-respiratory and infectious diseases) as well as with the adoption of unhealthy lifestyles and medical problems (including but not limited to obesity, cancer, liver disease, chronic lung disease, and cardiovascular disease) in adulthood (Flaherty et al., 2006; Lanier et al., 2010; Leeb et al., 2011; Shonkoff et al., 2012). Adverse childhood experiences may also lead to reduced academic (i.e., failing in school) and post-academic (i.e., unemployment, poverty, and homelessness) success (Shonkoff et al., 2012). The experience of interpersonal distress during childhood is also correlated with depression, aggression, and drug misuse (e.g., cigarette smoking, alcohol consumption, and illegal drug use) later in life (DeFronzo and Pawlak, 1993; Anda et al., 1999; Nation and Heflinger, 2006; Leeb et al., 2011; Shonkoff et al., 2012).

It has been shown that abnormal social attachments and deficits in social interactions are core features of most psychopathologies, including autism spectrum disorder, social phobia, obsessive-compulsive disorder, post-traumatic stress disorder, and borderline personality disorder (reivewed by Bartz and Hollander, 2006). As social bonds play important roles in the psychological, physiological, and behavioral functioning and deficits in social bonding are core features of various psychopathologies, the research investigating the neural correlates of social bond formation is highly important. Research has provided ample evidence to suggest that social interactions (including the bond observed between socio-sexual partners and between parents and offspring, as well as trust in social interactions) seem to share similar neural mechanism (e.g., Curley and Keverne, 2005).

The structurally similar neuropeptides, oxytocin (OT), and arginine vasopressin (AVP), have repeatedly been implicated in the regulation of social cognition and behavior (including social recognition, affiliation, aggression, pair bonding, as well as parental behavior) (Lim and Young, 2006; Donaldson and Young, 2008; Heinrichs et al., 2009). OT and AVP may act as neurotransmitters, if released centrally via the hypothalamus (namely the paraventricular and supraoptic nuclei of the hypothalamus), or as neurohormones, if released peripherally via the pituitary gland (Ludwig and Leng, 2006; Debiec, 2007). It is believed that the behavioral effects of OT and AVP are primarily due to the central release from the parvocellular neurons of the paraventricular nucleus (Debiec, 2007). It should be noted that various other neurochemicals, including dopamine, endogenous opioids, adrenocorticotropic hormone (ACTH), gamma-aminobutyric acid (GABA), may also play a role in the regulation of social cognition and behavior (reviewed by Carter et al., 1995).

The following review will focus on the role of the neuropeptides OT and AVP in mediating social attachments between partners and between parents and their offspring. Such selective and enduring relationships are complex and likely depend on distinct processes, including approach behaviors, formation of social recognition memory, and subsequently the formation and maintenance of the bond. Therefore, we will discuss the role of OT and AVP in partner bonds and parent-offspring bonds focusing in particular on these aspects of social attachments. In addition, the influence of OT and AVP on relationships between close individuals, particularly trust between peers, will be discussed. We will focus exclusively on the mammalian literature, including evidence from studies in rodents, non-human primates, and humans. Finally, we will discuss how our current knowledge may shape future research to better understand the neural correlates of social bonding and its implications in pathologies.

## Social bonds between partners in a mating pair

Romantic or passionate love, the intense attraction between mates, is an essential component of human social behavior and often precedes the formation of enduring preferential associations between two sexual partners. Such pair bonds are a type of social bond that occurs in nearly all human societies. While there are various definitions, a pair bond is commonly defined, across species, as an enduring preferential association between two sexually mature adults. The established social bond—a complex social behavior—is characterized by affiliative behaviors, copulation, and the selective preference toward the partner over a stranger (partner preference) (Gubernick, 1994). The bond is also often associated with mate guarding and bi-parental care of the offspring (Kleiman, 1977; Buss, 1988; Fraley et al., 2005). The significance of positive, enduring social bonds between human partners has been documented cross-culturally. For example, stable marital relationships are positively correlated with higher life expectancy compared to the life expectancy displayed by individuals who are single (House et al., 1988; Lillard and Waite, 1995; Drefahl, 2012). There are also strong positive associations between high levels of marital satisfaction and immune function as well as cardiovascular health (Kiecolt-Glaser and Newton, 2001; Kiecolt-Glaser et al., 2010). As enduring social bonds between partners are important to the psychological, physiological, and behavioral well-being (for review see Baumeister and Leary, 1995), considerable efforts have been made to identify the neurobiological mechanisms underlying pair bond formation. It should be mentioned that traditional laboratory rodents usually do not display pair bond formation, a behavioral characteristic that is displayed by less than 5% of mammalian species (Kleiman, 1977; Dewsbury, 1988). Therefore, various nontraditional animal models, which display pair bond formation—including marmosets (*Callithrix penicillata*), titi monkeys (*Callicebus cupreus*), California mice (*Peromyscus californicus*), and prairie voles (*Microtus ochrogaster*)—have emerged as better alternative animal models to study the neural correlates of pair bond formation (Carter et al., 1995; Bester-Meredith et al., 1999; Bales et al., 2007; de Jong et al., 2009; Smith et al., 2010). The nonapeptides, OT, and AVP, have been implicated in the regulation of social recognition and the display of prosocial/proximity-seeking behaviors—processes important for the formation of social bonds—as well as the formation of the bond itself (reviewed by Insel, 1992; Dluzen et al., 2001; Young, 2002; Bielsky and Young, 2004; Winslow and Insel, 2004; Wacker and Ludwig, 2012).

Using neuropharmacological manipulations, results suggest that OT and AVP play a role in pair bond formation across various mammalian species, including humans. Social recognition, an essential process to allow the formation of social bonds (Carter et al., 1995), allows members in a mating pair to distinguish between the familiar partner and an unfamiliar stranger. Several studies have implicated OT and AVP in mediating social recognition (reviewed by Engelmann et al., 1996; Dluzen et al., 2001; Bielsky and Young, 2004; Winslow and Insel, 2004; Wacker and Ludwig, 2012). In particular, the peripheral, central, and site-specific (e.g., into the lateral septum, main olfactory bulbs, and medial preoptic area) administration of OT facilitates social recognition in rats by increasing the retention time in a social discrimination test (Popik and van Ree, 1991; Popik et al., 1992a,b; Dluzen et al., 1998a,b, 2000), whereas the administration of a selective OT receptor, OTR, antagonist blocks short-term social recognition in rats and mice (Popik and van Ree, 1991; Engelmann et al., 1998; Dluzen et al., 2000; Ferguson et al., 2000). OT administration is also able to reverse the deficits in social memory observed in OT knockout mice (Ferguson et al., 2000, 2001). Similarly to OT, peripheral, central, and site-specific (e.g., into the lateral septum and main olfactory bulbs) AVP administration increases social recognition memory in rats (Dantzer et al., 1987, 1988; Le Moal et al., 1987; Popik et al., 1992b; Dluzen et al., 1998a,b) and rescues the social recognition impairment observed in Brattleboro rats, which carry a genetic mutation causing the lack of endogenous AVP (Engelmann and Landgraf, 1994). On the contrary, the peripheral, central, or site-specific (e.g., into the hippocampus, lateral septum, or main olfactory bulbs) administration of a non-selective AVP receptor antagonist, selective AVP receptor (V1aR) antagonist, or V1aR antisense oligodeoxynucleotide impairs social recognition memory in mice and rats (Dantzer et al., 1987, 1988; Bluthe et al., 1990; Popik et al., 1992b; Engelmann and Landgraf, 1994; Landgraf et al., 1995; van Wimersma Greidanus and Maigret, 1996; Everts and Koolhaas, 1997, 1999; Bielsky et al., 2005; Tobin et al., 2010). Rats treated site-specifically into the lateral septum with antisense oligodeoxynucleotides to the mRNA of V1aR investigate a familiar and a novel juvenile similarly, while rats treated with vehicle or scrambled-antisense oligodeoxynucleotide investigate the familiar significantly less than the novel juvenile—an indication of impaired social recognition (Landgraf et al., 1995). Similarly, the treatment with a V1aR antagonist, but not saline, into the lateral septum blocks the reduction in social investigation between a familiar and novel juveniles in rats (Dantzer et al., 1988; Popik et al., 1992b; Engelmann and Landgraf, 1994; Everts and Koolhaas, 1997, 1999; Bielsky et al., 2005). Site-specific treatment with anti-AVP serum into the ventral as well as dorsal hippocampus prevents the reduction of social investigation time between initial and second encounter of a juvenile as observed in control rats (van Wimersma Greidanus and Maigret, 1996). Treatment with an AVP antagonist into the main olfactory bulb inhibits the discrimination of a familiar vs. novel juvenile (Tobin et al., 2010). In extension to the memory-enhancing effects of OT in rats, intranasal OT administration also enhances the short- and long-term memory performance of both men and women in a face recognition test (Savaskan et al., 2008; Rimmele et al., 2009). However, it should be noted that additional research is necessary to interpret the effects of peripheral manipulations of the OT and AVP systems in affecting social recognition. In particular, research needs to systematically assess whether extrahypothalamic OT and AVP neurons are part of the neuronal circuit that is involved in social memory.

Further evidence for the involvement of OT and AVP in social bonds comes from neuropharmacological studies showing that OT and AVP play a role in the regulation of prosocial and affiliative behaviors. Such regulation is another essential component for the formation of a pair bond. For example, female prairie voles display higher levels of social behavior (including more side-by-side contact and less aggression) following a peripheral or central OT administration (Witt et al., 1990). Similarly, chronic OT administration increases social interactions of male rats with a female (as shown by an increase in the amount of side-by-side contact displayed and the duration of anogenital investigation) (Witt et al., 1992). The central OT administration increases approach, affiliative grooming (an important component of social interactions in various primate species) (Hinde and Berman, 1983), and huddle behavior toward a female in subordinate, but not dominant, male squirrel monkeys (*Saimiri sciureus*) (Winslow and Insel, 1991). In monogamous marmosets (*C. penicillata*), OT treatment increases the frequency of contact behavior, decreases the partner approach latency, and increases food sharing with the partner; whereas the treatment with a selective OTR antagonist reduces the frequency of contact behavior and decreased food sharing with the partner (Smith et al., 2010). Using adeno-viral vector-mediated (AAV) gene transfer, mice, rats, and meadow voles (*Microtus pennsylvanicus*, a promiscuous and nonsocial rodent species) can be engineered to express a similar V1aR pattern as the prairie vole. In turn, this receptor expression pattern in male mice, rats, and meadow voles significantly increases the affiliative behavior toward conspecific females (Young et al., 1999; Landgraf et al., 2003; Lim et al., 2004). Furthermore, intranasal AVP administration results in an increase in affiliative behavior (e.g., higher frequency of contact time) in male titi monkeys (*C. cupreus*) toward their female partner (Jarcho et al., 2011). Recent studies in humans have also provided evidence for the involvement of OT in the regulation of prosocial and affiliative behaviors. Using the Relationship Closeness Induction Task (a social psychology method used to introduce strangers to each other), intranasal OT administration increases conversational intimacy in women, but not men (Liu et al., 2012). Conversational intimacy is an index of human social approach behavior (Liu et al., 2012). Furthermore, intranasal OT treatment increases positive communication behavior in relation to negative behavior during a couple conflict discussion (Ditzen et al., 2009). In addition, researchers comparing plasma OT levels between new lovers (3 months after initiation of their romantic relationship) and non-attached singles, found that OT levels do not differ between men and women, are individually stable, and are higher in new lovers than singles (Schneiderman et al., 2012). Interestingly, the OT levels were positively correlated with the level of interactive reciprocity (i.e., affectionate touch) (Schneiderman et al., 2012). Similarly to plasma OT levels, plasma AVP levels also correlate positively with social functioning. In particular, higher levels of plasma AVP levels are associated with fewer negative marital interactions (Gouin et al., 2012).

Neuropharmacological manipulations of the OT and AVP systems, in particular using the socially monogamous prairie vole as an ideal model to study the neural correlates of pair bond formation between socio-sexual partners (Dewsbury, 1975; Williams et al., 1992b), provide further evidence that OT and AVP play an essential role in pair bond formation. Central or site-specific (e.g., into the nucleus accumbens) OT treatment, compared to artificial cerebrospinal fluid treatment, results in the formation of partner preference, an index of bond formation (Insel and Hulihan, 1995), in female prairie voles (Williams et al., 1992a; Insel and Hulihan, 1995; Cho et al., 1999; Liu and Wang, 2003). While mating (and/or cohabitation) is an essential component for pair bond formation (Getz et al., 1981; Williams et al., 1992b), OT-induced partner preference formation occurs even in the absence of mating. However, when females are pre-treated with a selective OTR antagonist, the OT treatment does not result in a partner preference (Williams et al., 1992a; Insel and Hulihan, 1995; Cho et al., 1999). In addition, a selective OTR antagonist given centrally before mating or in combination with OT also inhibits the formation of a partner preference in female prairie voles without affecting mating *per se* (Insel and Hulihan, 1995; Cho et al., 1999). The blockade of partner preference formation due to a selective OTR antagonist indicates that OT is working via an OTR-mediated mechanism. Interestingly, the effect of central OT seems to be gender-specific as OT administration does not result in partner preference formation and selective OTR antagonist administration does not block partner preference formation in males (Williams et al., 1992a; Winslow et al., 1993; Insel and Hulihan, 1995; but see Cho et al., 1999). Similarly to OT, AVP also plays a role in the formation of partner preference in prairie voles. In particular, central or site-specific (e.g., ventral pallidum) AVP treatment increases partner preference formation in male prairie voles in the absence of mating, while the treatment with a non-selective AVP receptor antagonist or a selective V1aR antagonist blocks the formation of partner preference formation (Winslow et al., 1993; Insel and Winslow, 1998; Cho et al., 1999; Lim and Young, 2004). It should be noted that such AVP-induced partner preference formation in female prairie voles is only observed when using a high dose of AVP (Insel, 1997; Cho et al., 1999).

Studies using genetic manipulations have also provided evidence indicating that social bonds (including the underlying components of social recognition, social affiliation, and, in turn, pair bond formation) are regulated in part by the neuropeptides, OT and AVP. For example, mice and Brattleboro rats that are null-mutants of the OT or AVP gene, respectively, show deficits in a social recognition task; but social recognition can be restored by OT and AVP administration (Ferguson et al., 2000, 2001; Winslow and Insel, 2002; Choleris et al., 2006; Crawley et al., 2007; Macbeth et al., 2009). In particular, OT treatment site-specifically into the medial amygdala is able to restore social recognition in OT knockout mice, indicating that amygdala OT plays an essential role in social recognition (Ferguson et al., 2001). Profound impairments in social recognition are also observed in OTR and V1aR knockout mouse species (Bielsky et al., 2004, 2005; Takayanagi et al., 2005; Macbeth et al., 2009). The re-expression of V1aR (using AAV) in the lateral septum of V1aR knockout mice can fully restore social recognition (Bielsky et al., 2005). In addition, the overexpression of the V1aR in the lateral septum of wild-type mice and rats can potentiate social recognition (Landgraf et al., 2003; Bielsky et al., 2005). Interestingly, CD38 knockout mice also show deficits in social recognition. CD38 knockout mice lack CD38, a type-II transmembrane protein. This protein is involved in the mobilization of intracellular Ca^2+^ via activation of cyclic ADP ribose (cADPR), a cellular messenger for calcium signaling (Jin et al., 2007). In turn, intracellular calcium signaling plays a key role in the central release of OT (Lopatina et al., 2013). Consequently, CD38 knockout mice have reduced levels of OT and display impairments in social recognition (Jin et al., 2007; Lopatina et al., 2013).

Genetic tools have also shown the involvement of OT and AVP in the display of prosocial and affiliative behaviors. Increasing the V1aR density via AAV-mediated gene transfer increases social affiliation and also shortens the length of cohabitation necessary for partner preference formation in male prairie voles (Pitkow et al., 2001). Interestingly, transgenic mice, expressing V1aR in a pattern resembling prairie vole V1aR expression, show an increase in affiliative behaviors toward females (including olfactory investigation and grooming) (Young et al., 1999). Prosocial behavior is also potentiated in male rats and prairie voles in response to the overexpression of V1aR in the lateral septum and ventral pallidum, respectively, (Pitkow et al., 2001; Landgraf et al., 2003). Furthermore, a recent study in humans indicates that single nucleotide polymorphisms (SNPs) of the OTR gene, namely the variants rs13316193, rs2254298, rs1042778, rs2268494, and rs226849, are associated with impairments in empathic communication (including less support-giving interactions and affective congruence) at the beginning of a romantic relationship. These impairments suggest that OT mediates human affiliative/prosocial behavior as an essential component of pair bond formation (Schneiderman et al., 2013).

Using genetic tools, researchers have also acquired evidence that OT and AVP play a role in the formation of the pair bond. For example, the overexpression of the V1aR in the ventral pallidum results in a strong partner preference formation in male prairie voles even in the absence of mating (Pitkow et al., 2001). Similarly, AAV-mediated V1aR gene transfer into the ventral forebrain of the promiscuous meadow vole (*M. pennsylvanicus*) enhances partner preference formation, a behavior that is not typically displayed by meadow voles (Lim et al., 2004). On the contrary, the knockdown of V1aR in the ventral pallidum of male prairie voles causes a deficit in partner preference formation (Barrett et al., 2013). Lastly, a number of recent findings suggest that OT and AVP may also play a role in the regulation of social bonding in humans. In particular, researchers assessed whether genetic variations in the OT and AVP receptor genes are associated with pair bonding and social behavior. The SNP in the OTR gene (rs7632287) seems to be associated with pair bonding in women. Women carrying one or two A-alleles score lower on the partner bonding scale and the relationship quality survey as well as are more likely to report martial problems than woman carrying two G alleles (Walum et al., 2012). The variation in the V1aR gene, *AVPR1A*, also seems to contribute to differences in human pair bonding. The allelic variant, RS3 334, is associated in men, but not women, with a lower bonding quality with the partner (characterized by lower scores on the partner bonding scale and a greater likelihood of reporting martial problems) (Walum et al., 2008). Interestingly, the association between the gene variants of the OTR and *AVPR1A* and the quality of pair bonding is also detected in the partner's perception of marital satisfaction (Walum et al., 2008, 2012).

## Parental bonding

Most mammalian offspring, including human children, are dependent on parental care for survival (Bowlby, 1951; Ainsworth, 1979; Nowak et al., 2000). For the young, parental care is essential to their individual survival; for the parent, parental care—characterized by one or both parents providing offspring with nutrition, warmth, shelter, as well as predatory protection—is an essential component of mammalian fitness (de Jong et al., 2009). Positive, enduring bonds between parents and their children play a significant role for the psychological, physiological, and behavioral functions of both parents and children (Silverstein and Bengtson, 1991; Graziano et al., 2009). For example, the presence of positive, secure attachments between parents and children provide protection against depression and anxiety in the child as well as lead to higher levels of child academic success (Bögels and Phares, 2008; Sarkadi et al., 2008; Graziano et al., 2009). On the contrary, the lack of positive, enduring parental bonding is associated with poor child health at an early age (e.g., increased risk for asthma, as well as cardio-respiratory and infectious diseases) as well as with the adoption of unhealthy lifestyles and medical problems (including but not limited to obesity, cancer, liver disease, chronic lung disease, and cardiovascular disease) in adulthood (Flaherty et al., 2006; Lanier et al., 2010; Leeb et al., 2011; Shonkoff et al., 2012). In addition, the lack of positive parental bonds can also lead to reduced academic success, depression, aggression, and drug misuse (DeFronzo and Pawlak, 1993; Anda et al., 1999; Nation and Heflinger, 2006; Leeb et al., 2011; Shonkoff et al., 2012). As positive social bonds between parents and their children are essential for the psychological, physiological, and behavioral well-being of both parents and child, considerable efforts have been expanded to identify the neurobiological mechanisms underlying parental bond formation. Various animal models, primarily rats and sheep, have been used to study the neural correlates of maternal behavior (Kendrick, 2000). Very little is known about paternal behavior, which is relatively rare among mammals and is displayed by less than 5% of mammalian species that are monogamous, including humans (Kleiman, 1977). More recently, untraditional animal models including the male California mouse and the male prairie vole have emerged to study the neurobiological mechanism underlying paternal behavior (Kentner et al., 2010). Parental bonding depends on the approach (including the display of prosocial behavior), social recognition of the offspring, and subsequently the formation of a selective and enduring bond between parent and offspring. This complex social behavior seems to be regulated in part by OT and AVP (Kendrick, 2000; de Jong et al., 2009; Bosch and Neumann, 2012; Nagasawa et al., 2012).

Through neuropharmacological manipulations, evidence has accumulated suggesting that OT and AVP play a role in parental bond formation across various different mammalian species, including humans. The initiation of parental behavior depends on the approach and display of prosocial behavior, a process that seems to be facilitated in part by the neuropeptides, OT, and AVP. Central OT administration reduces the latency of female rats to show maternal behavior when exposed to pups (Pedersen and Prange, 1979; Pedersen et al., 1982; Fahrbach et al., 1984) and increases maternal behavior in mice (McCarthy, 1990). The central administration of the OTR agonist, Thr4, Fly7-OT, has a similar potency to OT in stimulating maternal behavior (Kendrick, 2000). In addition, central, but not peripheral, OT administration enhances the interest of nulliparous ewes toward newborns, increases the display of maternal behavior, and induces maternal responsiveness even without the experience of natural parturition (Kendrick et al., 1987; Keverne and Kendrick, 1992; Levy et al., 1992). Administration of an OTR antagonist site-specifically into the nucleus accumbens inhibits “spontaneous” maternal behavior in sexually naïve female prairie voles, which provides further evidence that OT may play a role in maternal behavior (Olazabal and Young, 2006). Furthermore, a correlational study in humans suggests the involvement of OT in maternal behavior. In particular, an increase in plasma OT level from the first to the third trimester is linked with maternal bonding to the fetus during the third trimester (Levine et al., 2007). Additionally, high levels of OT in the first trimester predict the amount of postpartum maternal behavior (including gaze, positive affect, and “motherese” vocalizations) (Feldman et al., 2007). Similarly to OT, central AVP administration also induces maternal behavior in rats (Pedersen et al., 1982). Chronic central AVP administration or overexpression of V1aR in the medial preoptic area (using AAV) increases arched back nursing behavior in female rats (Bosch and Neumann, 2008). On the contrary, antagonizing OT (via the administration of a selective OTR antagonist, antisera to OT, or an analog OT antagonist) centrally or site-specifically (e.g., into the medial preoptic area, olfactory bulb, or ventral tegmental area) can delay or block the onset of maternal behavior in rats (Fahrbach et al., 1985; Pedersen et al., 1985, 1994; van Leengoed et al., 1987; Yu et al., 1996) as well as reduce the display of arched back nursing behavior in rats (Bosch and Neumann, 2008). Similarly, the central or site-specific (e.g., into the medial preoptic area) administration of AVP antisera, V1aR antisense oligodeoxynucleotide, or V1aR antagonist decreases maternal behavior (Pedersen et al., 1985, 1994; Bosch and Neumann, 2008). Unfortunately due to the lack of an appropriate animal model to study the neural correlates of paternal behavior, only recently have researchers started to acquire knowledge about the underlying neural mechanisms. Congruent with the OT and AVP regulation of maternal affiliative behaviors toward pups (reviewed by Bosch and Neumann, 2012), these neuropeptides may also play a role in the onset of paternal affiliative behaviors toward pups. For example, parentally naïve male California mice show higher levels of OT when housed with a pregnant female than sexually naïve males or new fathers (Gubernick et al., 1995). There is also a transient increase of OT in male prairie voles that have been exposed to pups (Kenkel et al., 2012). Central treatment with OT, but not vehicle, in male common marmosets reduces the frequency of refusal of food transfer to the infant (Saito and Nakamura, 2011). As food transfer from mother or father to infant is considered caretaking behavior, the OT-induced reduction in food transfer refusal indicates an increase in parental behavior. There is evidence to suggest that AVP may also be involved. In early prairie vole studies, new fathers, compared to sexually naïve males, showed an increase in AVP mRNA expression in the bed nucleus of the stria terminalis (Wang et al., 1994b), but showed a decreased density of AVP-immunoreactive fibers in the lateral septum (Bamshad et al., 1993). These changes in the AVP system suggest an increased AVP release in the lateral septum, which is associated with the enhanced display of paternal behavior in the father voles (Wang et al., 1998). In a pharmacological experiment, the administration of AVP into the lateral septum of sexually naïve male prairie voles enhances paternal behavior (including grooming, crouching over, and contacting pups); whereas the administration of an AVP receptor antagonist inhibits paternal behavior (Wang et al., 1994a; Bales et al., 2004). These data demonstrate the functional role of central AVP in paternal behavior. Interestingly, central AVP treatment can also induce facultative paternal behavior in the typically non-paternal meadow vole, which can be blocked by the central treatment with a selective V1aR antagonist (Parker and Lee, 2001). In addition to the evidence that AVP plays a role in mediating paternal behavior in rodents, a study using male marmosets (*C. jacchus*) showed that fathers, both first time and experienced ones, have greater dendritic spine density on pyramidal neurons in the prefrontal cortex as compared to non-father marmosets (Kozorovitskiy et al., 2006). Most interestingly, the density of AVP V1aR and the proportion of V1aR-labeled dendritic spines increased significantly in fathers compared to non-fathers (Kozorovitskiy et al., 2006). These data indicate that AVP is involved in the neural reorganization associated with the experience of fatherhood in a bi-parental primate species. There is also evidence to suggest that OT and AVP interact in the regulation of paternal care. Bales et al. (2004) noted that only the combined treatment with OT and AVP antagonists fully reduces paternal care (i.e., inhibit kyphosis, pup approach, nonkyphotic contact, licking/grooming, and retrieving) in naïve male prairie voles. Furthermore, there is evidence indicating that OT and AVP may play a role in human parental behavior. In particular, the OT concentration in the mother's cerebrospinal fluid is significantly increased following birth (Takagi et al., 1985) and plasma OT levels after birth are correlated with maternal behavior (including gaze, vocalizations, positive affect, and affectionate touch) (Feldman et al., 2007). In addition, plasma OT levels show intra-individual stability across the first 6 months of parenthood and are positively correlated with maternal and paternal behavior (including “motherese” vocalizations, positive affect, and affectionate touch) (Feldman et al., 2010, 2011; Gordon et al., 2010a,c; Apter-Levi et al., 2013). Similarly, plasma AVP levels predict maternal and paternal behavior (particularly joint attention to inanimate objects and stimulatory contact) to their infant (Apter-Levi et al., 2013). Furthermore, triadic synchrony (defined as moments in which physical proximity and affectionate touch is displayed between parents and between parent and infant) is correlated with plasma OT (Gordon et al., 2010b).

Pharmacological manipulations suggest that the nonapeptides, OT and AVP, not only play a role in the initiation of maternal behavior, but also have an important role in mediating social recognition, another vital component for parental bonding. Indeed, social recognition between mothers and their offspring has been shown in various species including sheep (Ferreira et al., 2000), goats (Poindron et al., 2003), Southern pig-tailed macaques (*Macaca nemestrina*) (Jensen, 1965), Barbary macaques (*Macaca sylvanus*) (Hammerschmidt and Fischer, 1998), rhesus macaques (*Macaca mulatta*) (Jovanovic et al., 2000), and even rodents (Ostermeyer and Elwood, 1983). Using the sheep as a model to study the neural correlates for maternal bonding, OT has been shown to play a critical role in the olfactory recognition of the lamb (Kendrick et al., 1997). Once a ewe has formed such social recognition memory, it can distinguish its lamb from others and will reject all lambs other than their own—indicating the formation of a mother-offspring bond (Kendrick, 2000). Nephew and Bridges (2008) showed that the site-specific administration (into the medial amygdala, but not the lateral ventricles) of a V1aR antagonist blocks social recognition in female rats. While only very few studies have been performed to assess parent-offspring bonding in humans, it is still plausible to hypothesize that such parent-child bonding does occur in humans. In particular, human mothers are capable of recognizing their own infant within 30 min after birth (Porter et al., 1983).

Additional support of the involvement of OT and AVP in the initiation of parental behavior, comes from genetic studies. OTR and paternally expressed gene 3 (*peg3*) knockout mice display deficits in maternal behavior including nurturing of pups, nest building, pup retrieval, and crouching behavior (Li et al., 1999; Ragnauth et al., 2005; Pedersen et al., 2006; Champagne et al., 2009). The gene *peg3* codes for a large zinc finger protein, a transcription factor that has been implicated in p53-mediated apoptosis (Deng and Wu, 2000; Relaix et al., 2000), which may account for the reduction in hypothalamic OT neurons and OTR in *peg3* knockout mice (Relaix et al., 1998; Li et al., 1999). Further, Brattleboro rats, which are genetically incapable of producing AVP, also show deficits in maternal care evidenced by a lower survival rate of offspring (Wideman and Murphy, 1990). Female and male CD38 knockout mice, which exhibit low central OT levels in comparison to wild-type mice, as mentioned above, also display deficits in parental behavior (i.e., lower rate of pup retrieval and crouching over pups) (Jin et al., 2007; Akther et al., 2013). The re-expression of CD38 (using AAV) in the nucleus accumbens and OT administration in CD38 knockout mice can restore paternal behavior (including retrieval, pup grooming, crouching, and huddling) (Akther et al., 2013). In addition, the SNP of the prairie vole V1aR gene (*avpr1a*) is associated with parental behavior. Specifically, male prairie voles with the allele coding for a long *avpr1a* microsatellite display higher levels of licking/grooming behavior than male prairie voles with the short *avpr1a* microsatellite (Hammock and Young, 2005). Microsatellites, or short tandem repeats, consisting of one to six nucleotides, which are repeated several times and therefore contribute to genetic variation (Guichoux et al., 2011).

In humans, SNPs in the genes for the OTR and CD38 have been implicated in parental behavior. In particular, the OTR gene alleles rs2254298 and rs1042778 as well as the CD38 gene allele rs3796863 are associated with lower plasma OT levels, and in turn, lower rates of parental care (i.e., shorter duration of parent-infant gaze synchrony and less amount of touch toward the infant) (Feldman et al., 2012). Future studies are needed to address whether genetic manipulations can show the involvement of OT and AVP in the paternal recognition of offspring and the parental-offspring bond formation *per se*.

## Interpersonal bonding

Similarly to the importance of attachment between partners of a mating pair and between parents and their offspring, mutually cooperative interactions involving the display of trust are an essential aspect of human society (Luhmann, 1979; Coleman, 1990). Trust is essential in love, families, and friendships. While a lot of effort has been expanded to investigate the neural correlates of social behavior, the neural mechanisms underlying trust are just beginning to be examined. Interestingly, it appears that the neuropeptide OT is not only involved in facilitating various social behaviors, such as pair bonding (Dluzen and Carter, 1979; Insel and Shapiro, 1992; Insel, 1997; Carter, 1998) and maternal attachment (Insel and Young, 2001; Bosch and Neumann, 2012), but has also been implicated in playing a role in trust (reviewed by De Dreu et al., 2010).

Research on the role of OT in regulating the display of trust revealed that OT levels increase in response to the display of intentional trust in a trust game with real monetary stakes. In particular, the level of peripheral OT (as assessed using blood) increases in subjects who received an intentional trust signal (Zak et al., 2004, 2005). However, it should be noted that currently the relationship between peripheral OT and central OT in humans is still unknown. Nevertheless, the findings of increased peripheral OT in response to intentional trust should be considered in the light of the hyperfunctional magnetic resonance imaging (hyperfMRI) study by Krueger et al. (2007). The authors report significant activity in the septal area when subjects display trust intentionally. Based on evidence from rodent studies, the septal area is involved in the regulation of social behavior (including pair bonding and paternal behavior) (Young et al., 2005; Skuse and Gallagher, 2009). In addition, the lateral septum via its connections to the nucleus accumbens may also represent a potential link between social behavior and reward circuitries (Young et al., 2005; Skuse and Gallagher, 2009). Furthermore, intranasal administration of OT increases interpersonal trust (see review by Van Ijzendoorn and Bakermans-Kranenburg, 2012), without affecting non-social risk-taking behaviors (Kosfeld et al., 2005). Specifically, 13 out of 29 subjects who received nasal spray containing OT showed the maximal trust level (i.e., sending all the money to another person in the “*trust game*” with real monetary stakes), whereas only 6 out of 29 subjects who received placebo showed maximal trust (Kosfeld et al., 2005). It should be noted that OT-induced trust only increases in the absence of cues that the partner is untrustworthy (Mikolajczak et al., 2010a). Exogenous administration of OT also increases the trust displayed toward one's own group (in-group trust). Using the intergroup prisoner's dilemma-maximizing difference game, it has been shown that intranasal OT increases in-group trust as displayed by an increase in the (monetary) contributions to the in-group, without affecting out-group distrust (De Dreu et al., 2010). In addition to the trust-increasing effects of OT in a monetary scenario, exogenous OT also increases trust in non-monetary scenarios. It has been observed that intranasal OT decreases the perceived risk of betrayal and increased trust in a scenario involving intimate and confidential information (Mikolajczak et al., 2010b). In particular, 60% of the participants who received OT, compared to only 3% of participants who received placebo, left a letter containing intimate and confidential information unsealed (Mikolajczak et al., 2010b). In addition, intranasal OT increases a person's willingness to share emotions, without affecting level of talkativeness (Lane et al., 2013).

Additional evidence for the involvement of OT in the regulation of trust comes from genetic studies (reviewed by Donaldson and Young, 2008). It should be noted that trust behavior appears to be heritable, indicating that a specific gene may be responsible for the variations in trust observed across individuals (Cesarini et al., 2008). In particular, using genetic screening for the OTR gene and the “*trust game*” showed that the rs53576, rs1042778, rs2268490, and rs237887 SNPs of the OTR gene is associated with greater trusting behavior (Israel et al., 2009; Krueger et al., 2012). Similarly to OT, AVP seems to play a role in mediating trust as well. In particular, the *AVPR1A* promoter region length (rs3 microsatellite polymorphism) is associated with greater money allocation, suggesting greater trusting behavior, in the “*trust game*” (Knafo et al., 2008).

## Conclusion

Over the past decades, the understanding of the neurobiological mechanism underlying bonding behaviors has substantially increased. In particular, studies using various animal models have provided evidence of the involvement of the nonapeptides, OT and AVP, in the regulation of social bonding (including bonding between mates in a mating pair and bonding between parent and offspring) (see Table 1). In addition, experimental techniques (such as intranasal OT administration and gene sequencing) in humans have resulted in evidence suggesting that these nonapeptides may also play a role in the regulation of bonding in humans. However, it should be noted that other neurochemicals, such as prolactin, sex hormones, catecholamines, endogenous opiates, and GABA may also play a role in the regulation of social behaviors. Thus, future research is needed to determine the interplay of these various neurochemicals to regulate social bonding across various species. It is also of importance to mention that there are instances of sexual dimorphisms in the regulation of social bonding by OT and AVP (e.g., OT and AVP differently regulate social bonds in female and male prairie voles). Therefore, sex differences in the regulation of social bonding warrant further investigation.

**Table 1:** **Effects of pharmacological manipulations of OT and AVP on bonding behavior**.

**Type of bond**	**Treatment**	**Species**	**Effect**	**References**
**PAIR BOND**				
Social recognition	Peripheral OT	Rat	↑	Popik et al., 1992a
	Central OT	OT KO mouse	↑	Ferguson et al., 2000
	Site-specific OT (e.g., AMY, LS, MPOA, OB)	OT KO mouse, rat	↑	Popik and van Ree, 1991; Popik et al., 1992b; Dluzen et al., 1998a,b, 2000; Ferguson et al., 2001; Winslow and Insel, 2002
	Intranasal OT	Human	↑	Savaskan et al., 2008; Rimmele et al., 2009
	Central OTR-A	Rat, mouse	↓	Engelmann et al., 1998; Ferguson et al., 2000
	Site-specific OTR-A (e.g., MPOA, OB)	Rat	↓	Popik and van Ree, 1991; Dluzen et al., 2000
	Peripheral AVP	Rat	↑	Dantzer et al., 1987
	Central AVP	Rat	↑	Le Moal et al., 1987
	Site-specific AVP (e.g., LS, OB)	Brattleboro rat, rat, V1aR KO mouse	↑	Dantzer et al., 1988; Popik et al., 1992b; Engelmann and Landgraf, 1994; Dluzen et al., 1998a,b; Landgraf et al., 2003; Bielsky et al., 2005
	Central AVP-A	Rat	↓	Bluthe et al., 1990; van Wimersma Greidanus and Maigret, 1996
	Site-specific AVP antisense, AVP-A, or V1aR-A (e.g., hippocampus, LS, OB)	Mouse, rat	↓	Dantzer et al., 1988; Popik et al., 1992b; Engelmann and Landgraf, 1994; Landgraf et al., 1995; van Wimersma Greidanus and Maigret, 1996; Everts and Koolhaas, 1997, 1999; Bielsky et al., 2005; Tobin et al., 2010
Prosocial behavior	Peripheral OT	Rat, prairie vole	↑	Witt et al., 1990
	Central OT	Prairie vole, rat, marmoset, squirrel monkey	↑	Witt et al., 1990, 1992; Winslow and Insel, 1991; Smith et al., 2010
	Intranasal OT	Human	↑	Liu et al., 2012
	Central OTR-A	Marmoset	↓	Smith et al., 2010
	Intranasal AVP	Titi monkey	↑	Jarcho et al., 2011
Bond	Central OT	Prairie vole	↑	Williams et al., 1992a; Insel and Hulihan, 1995; Cho et al., 1999
	Site-specifc OT (e.g., NAcc)	Prairie vole	↑	Liu and Wang, 2003
	Central OTR-A	Prairie vole	↓	Insel and Hulihan, 1995; Cho et al., 1999
	Central AVP	Prairie vole	↑	Winslow et al., 1993
	Central AVPR-A or V1aR-A	Prairie vole	↓	Winslow et al., 1993; Insel and Winslow, 1998; Cho et al., 1999
	Site-specific V1aR-A (e.g., VP)	Prairie vole	↓	Lim and Young, 2004
**PARENTAL BOND**				
Social recognition	OT or OTR-A	na	na	na
	AVP or AVPR-A	na	na	na
Prosocial behavior	Central OT	♂ common marmoset, ♀ mice, ♀ rat, ♀ sheep	↑	Pedersen and Prange, 1979; Pedersen et al., 1982; Fahrbach et al., 1984; Kendrick et al., 1987; McCarthy, 1990; Keverne and Kendrick, 1992; Levy et al., 1992; Saito and Nakamura, 2011
	Site-specific OT (e.g., NAcc)	♀♂ CD38KO mouse	↑	Akther et al., 2013
	Central OTR-A	♀ rats	↓	Fahrbach et al., 1985; Pedersen et al., 1985; van Leengoed et al., 1987
	Site-specific OTR-A (e.g., MPOA, NAcc, OB, and VTA)	♀ prairie voles, ♀ rats	↓	Pedersen et al., 1994; Yu et al., 1996; Olazabal and Young, 2006
	Central AVP	♂ meadow voles, ♀ rats	↑	Pedersen et al., 1982; Parker and Lee, 2001
	Site-specific AVP (e.g., LS)	♂ prairie vole	↑	Wang et al., 1994a
	Central AVPR-A	♂ meadow voles, ♀ rats	↓	Pedersen et al., 1985; Parker and Lee, 2001; Bosch and Neumann, 2008
	Site-specific AVPR-A or V1aR-A (e.g., MPOA and LS)	Rats, ♂ prairie vole	↓	Pedersen et al., 1994; Wang et al., 1994a
	Central AVP-A and OTR-A	♂ prairie vole	↓	Bales et al., 2004
Bond	OT or OTR-A	na	na	na
	AVP or AVPR-A	na	na	na
**TRUST**	Intranasal OT	Human	↑	Kosfeld et al., 2005; De Dreu et al., 2010; Mikolajczak et al., 2010a,b

*Abbreviations used: AMY, amygdala; AVP, argenine vasopressin; AVP-A, non-selective AVP receptor antagonist; LS, lateral septum; MPOA, medial preoptic area; NAcc, nucleus accumbens; OB, olfacotry bulbs; OT, oxytocin, OTR-A, oxytocin receptor antagonist; VP, ventral pallidum; VTA, ventral tegmental area*.

### Conflict of interest statement

The authors declare that the research was conducted in the absence of any commercial or financial relationships that could be construed as a potential conflict of interest.

## Abbreviations

AAV: adeno-associated viral vector
AVP: arginine vasopressin
GABA: gamma-aminobutyric acid
OT: oxytocin
OTR: oxytocin receptor
V1aR: selective AVP receptor 1
SNP: single nucleotide polymorphism.
